# PR3-ANCA: A Promising Biomarker in Primary Sclerosing Cholangitis (PSC)

**DOI:** 10.1371/journal.pone.0112877

**Published:** 2014-11-14

**Authors:** Laura M. Stinton, Chelsea Bentow, Michael Mahler, Gary L. Norman, Bertus Eksteen, Andrew L. Mason, Gilaad G. Kaplan, Bjorn Lindkvist, Gideon M. Hirschfield, Piotr Milkiewicz, Angela Cheung, Harry L. A. Janssen, Marvin J. Fritzler

**Affiliations:** 1 Department of Medicine, University of Calgary, Calgary, Alberta, Canada; 2 Inova Diagnostics, Inc., San Diego, California, United States of America; 3 Department of Medicine, University of Alberta, Edmonton, Alberta, Canada; 4 Institute of Medicine, Sahlgrenska Academy, University of Gothenburg, Gothenburg, Sweden; 5 Centre for Liver Research, NIHR Biomedical Research Unit, University of Birmingham, Birmingham, United Kingdom; 6 Department of General, Transplant and Liver Surgery, Warsaw Medical University, Warsaw, Poland; 7 University Health Network, Division of Gastroenterology, Toronto Western Hospital, Toronto, Ontario, Canada; 8 Liver Research Laboratories, Pomeranian Medical University, Szczecin, Poland; Karolinska Institutet, Sweden

## Abstract

**Background and Aims:**

The only recognized biomarker for primary sclerosing cholangitis (PSC) is atypical anti-neutrophil cytoplasmic antibodies (aANCA), which, in addition to having low sensitivity and specificity, is an indirect immunofluorescence (IIF) test lacking the advantages of high throughput and objectivity. Recent reports have shown that antibodies to proteinase-3 (PR3-ANCA) might add diagnostic value in inflammatory bowel disease (IBD), specifically in ulcerative colitis (UC). As PSC is associated with IBD, the objective of this study was to evaluate the frequency and clinical significance of PR3-ANCA in a large cohort of patients.

**Methods:**

A total of 244 PSC and 254 control [autoimmune hepatitis (AIH), primary biliary cirrhosis (PBC), hepatitis C viral infection (HCV), hepatitis B viral infection (HBV), and healthy controls] sera and their clinical correlations were retrospectively analyzed for PR3-ANCA determined by ELISA and a new chemiluminescence immunoassay (CIA). Testing was also performed for aANCA by IIF.

**Results:**

When measured by CIA, PR3-ANCA was detected in 38.5% (94/244) of PSC patients compared to 10.6% (27/254) controls (p<0.0001). By ELISA, PR3-ANCA was detected in 23.4% (57/244) of PSC patients compared to 2.7% (6/254) controls (p<0.0001). PR3-ANCA in PSC patients was not associated with the presence or type of underlying IBD, and, in fact, it was more frequent in Crohn's disease (CD) patients with PSC than previously reported in CD alone. PR3-ANCA in PSC measured by CIA correlated with higher liver enzymes.

**Conclusion:**

PR3-ANCA is detected in a significant proportion of PSC patients compared to other liver diseases including PBC and AIH. PR3-ANCA is associated with higher liver enzyme levels in PSC, and is not solely related to underlying IBD.

## Introduction

Primary sclerosing cholangitis (PSC) is a chronic, cholestatic syndrome characterized by inflammation and fibrosis of the intra- and extra-hepatic bile ducts, leading to multifocal bile duct strictures. The clinical course and complications of PSC vary considerably, but usually follows a progressive course, ultimately leading to cirrhosis, hepatic failure, and in 10–20% of patients, cholangiocarcinoma. PSC is associated with inflammatory bowel disease (IBD) in 70–80% of cases, most commonly ulcerative colitis (UC) [1], [2].

The diagnosis of large duct PSC is based on a cholestatic elevation of liver enzymes and typical cholangiographic findings including bile duct irregularities with multiple strictures and segmental dilatations. A variety of autoantibodies have been observed in the sera of PSC patients, but none are disease-specific [3]. Anti-neutrophil cytoplasmic antibodies (ANCA), directed against various subcellular constituents of neutrophil or myeloid cells, have been reported in 65–95% of PSC patients [4]–[6]. ANCA are routinely detected by indirect immunofluorescence (IIF) assays using ethanol and formalin-fixed neutrophils [7]. The IIF ANCA staining pattern in PSC has been characterized as broad, non-homogeneous enhancement of the nuclear periphery combined with multiple intranuclear foci. This has been referred to as atypical ANCA (aANCA), anti-neutrophil nuclear antibodies (p-ANNA), or xANCA [7]. aANCA have been reported in the context of UC and PSC, but also in other liver diseases including autoimmune hepatitis (AIH), primary biliary cirrhosis (PBC), and viral and alcoholic hepatitis [2] [5], [6], [8], [9]. The other well known IIF ANCA patterns are cytoplasmic (cANCA) and perinuclear (pANCA). cANCA is largely attributed to the presence of autoantibodies targeting the serine protease proteinase-3 (PR3-ANCA), while pANCA is associated with antibodies directed against a number of antigens, including myeloperoxidase (MPO-ANCA), lactoferrin, lysozyme, azurocidin, elastase, cathepsin G, and bactericidal/permeability-increasing enzyme (BPI)[10], [11]. PR3-ANCA are an established marker for the diagnosis of small vessel vasculitis including granulomatosis with polyangiitis (GPA) (formerly Wegener's granulomatosus); MPO is the most frequently identified antigen in pANCA and is associated with crescentic glomerulonephritis, microscopic polyaangitis (MPA), and eosinophilic granulomatosis with polyangiitis (EGPA) (formerly Churg-Strauss syndrome) [7], [12]. aANCA has been widely investigated and putative targets include high mobility group non-histone, high mobility group chromosomal proteins HMG1/2 [13], beta-tubulin isotype 5 [14], [15], and DNA-bound lactoferrin[16].

In contrast to previously reported data [17], more recent studies indicate that PR3-ANCA are detected in a significant proportion of patients with IBD, specifically UC [18], [19]. This is particularly true when PR3-ANCA are detected by capture or anchor immunoassays, possibly because they bind conformational epitopes that are not available for binding in conventional enzyme-linked immunosorbent assays (ELISA) [18], [20]. PR3-ANCA measured by conventional ELISA has previously been reported in PSC, however the prevalence has ranged from 4–44% [3], [21]. The goal of this multi-centre international study was to evaluate the frequency of PR3-ANCA in PSC patients as measured by ELISA and a new chemiluminescence immunoassay (CIA) and to determine clinical correlations with PR3-ANCA. The utility of PR3-ANCA to aid in the diagnosis of PSC was compared to aANCA detected by IIF, while the diagnostic specificity of these assays was assessed in PSC and compared to other liver diseases including those of autoimmune etiology [primary biliary cirrhosis (PBC), autoimmune hepatitis (AIH) and overlap syndromes of AIH-PBC, AIH-PSC]. Finally, the use of PR3-ANCA and aANCA IIF in PSC and IBD was assessed.

## Materials and Methods

### Patient Samples

A total of 498 patient biobanked sera were analysed in this study. Serum samples from 222 PSC patients and 22 AIH-PSC originating from 5 clinical centres (Center I: Sahlgrenska University Hospital, Gothenburg, Sweden, n = 114; Center II: University Health Network, Toronto Western Hospital, Toronto, Ontario, Canada, n = 59; Center III: University of Calgary, Calgary, Alberta, Canada (Including the Alberta IBD Consortium), n = 27); Center IV: University of Alberta Hospital, Edmonton, Alberta, Canada, n = 26; Center V: Pomeranian Medical University, Szczecin, Poland, n = 18). A diagnosis of PSC was confirmed by clinical experts in the field based on the following criteria: chronically elevated liver enzymes; standard cholangiographic (magnetic resonance cholangiography [MRCP] or endoscopic retrograde cholangiography [ERCP]) bile duct changes with multifocal strictures and segmental dilatations and/or liver biopsy consistent with PSC, and exclusion of secondary cause of cholangitis. Small duct PSC was defined as a liver biopsy with histopathology consistent with PSC and a normal ERCP or MRCP. Clinical data from the PSC patients was collected when available and included age, sex, disease duration, type of PSC including small-duct PSC, presence of cholangiocarcinoma, cirrhosis, ascites, hepatic encephalopathy, esophageal varices, use of ursodeoxycholic acid (UDCA), and laboratory measurements (alanine aminotransferase (ALT), aspartate aminotransferase (AST), alkaline phosphatase (ALP), total bilirubin, international normalized ratio (INR), hemoglobin, platelet count, and creatinine). All clinical information correlated with the date of serum collection. Each site also provided the IBD status of the PSC patients. The diagnosis of IBD was made by experts in the field based on established diagnostic criteria including a combination of clinical, endoscopic, histological and serological results. Patients with PSC who were not evaluated for IBD were excluded. 254 serum samples from patients with various hepatic diseases were also included as pathological controls (65 AIH, 81 PBC, 10 AIH-PBC, 18 hepatitis C viral infection (HCV), 32 hepatitis B viral infection (HBV), 48 healthy controls) (Inova Diagnostics Inc., San Diego, CA, in-house control cohorts). Written consent was obtained from all patients in accordance with the project approved by the local ethical committees including the Conjoint Health Ethics Review Board at the University of Calgary and fulfilled the ethical guidelines established in the Declaration of Helsinki.

### Autoantibody Assays

PR3-ANCA positivity was determined by a novel CIA (QUANTA Flash PR3 on BIO-FLASH CIA, Inova Diagnostics Inc.) which uses native PR3 antigen coupled to paramagnetic beads. The PR3 CIA is designed around the BIO-FLASH instrument, containing a luminometer, as well as all the hardware and liquid handling accessories necessary to perform the assay. Native purified human PR3 is coated onto paramagnetic beads and assayed on the BIO-FLASH system as previously described [22]. The PR3 CIA utilizes a predefined lot specific Master Curve that is uploaded into the instrument through the reagent pack barcode. Based on the results of running two calibrators, an instrument specific Working Curve is created, which is used to calculate chemiluminescent units (CUs) for each serum. For the PR3 CIA IgA version, the isoluminol conjugated anti-human IgG was replaced by isoluminol conjugated anti-human IgA. All samples were tested by PR3 ELISA and by the PR3 CIA. The suggested cut-off value of 20 chemiluminescent units (CU) for the CIA as suggested by the manufacturer was utilized. Anti-PR3 was also determined by traditional ELISA (QUANTA Lite PR3, Inova Diagnostics, Inc.), which uses the same native antigen as the CIA system. ANCA was tested by IIF on formalin and ethanol-fixed neutrophil substrates (ANCA: Inova Diagnostics Inc.) and read on an Olympus CX31 microscope (Olympus America Inc., Melville, NY) by an experienced technologist.

### Statistical Methods

Statistical evaluation was performed using Analyse-it software (Version 2.03; Analyse-it Software, Ltd., Leeds, UK). Sensitivities, specificities and likelihood ratios were calculated where appropriate. Descriptive statistics and Fisher's exact test were used to compare groups where applicable. Mann-Whitney test and ANOVA were used for the comparison of continuous data. Receiver operator characteristic (ROC) curves were calculated to determine the ability of PR3-ANCA to discriminate PSC from the control groups. Mean titers of both the CIA and ELISA tests were used. Where appropriate, 95% confidence intervals (CI) were provided. For all statistical methods, p values <0.05 were considered significant.

## Results

### Prevalence of PR3-ANCA in PSC and liver disease controls

By CIA, 94/244 (38.5%) of PSC patients [82/222 (36.9%) PSC, 12/22 (54.5%) AIH-PSC] were positive for PR3-ANCA compared to 27/254 (10.6%) of controls (p<0.0001). By comparison the control sera had a remarkably lower frequency of PR3-ANCA: AIH 14/65 (21.5%), AIH-PBC 3/10 (30%), PBC 9/81 (11.1%), HBV 1/32 (3.1%), HCV 0/18, healthy controls 0/48. By ELISA, 57/244 (23.4%) of PSC patients [51/222 (23.0%) PSC, 6/22 (27.3%) AIH-PSC] were positive for PR3-ANCA compared to 6/254 (2.7%) of controls (p<0.0001) [AIH 5/65 (7.7%), AIH-PBC 0/10, PBC 0/81, HCV 0/18, HBV 0/32, healthy controls 1/48 (2.1%)]. aANCA by IIF were detected in 101/244 (41.4%) of PSC patients [89/222 (40.1%) PSC, 12/22 (54.5%) AIH-PSC] compared to 51/254 (20.1%) of controls (p<0.0001) [AIH 26/65 (40%), AIH-PBC 2/10 (20%), PBC 8/81 (9.9%), HCV 0/18, HBV 3/32 (9.4%), healthy controls 2/48 (4.2%)] (Table 1).

**Table 1:** Frequency of PR3-ANCA by CIA, ELISA, and aANCA in PSC vs. all controls, vs. AIH and vs. PBC.

	PSC			Controls			Relevance
	All PSC, n (%)	PSC, n (%)	AIH-PSC, n (%)	All Controls, n (%)	AIH, n (%)	PBC, (%)	p value
PR3-ANCA CIA	94/244 (38.5%)	82/222 (36.9%)	12/22 (54.5%)	27/254 (10.6%)	14/65 (21.5%)	12/91 (13.2%)	<0.0001^a^
							0.014 b
							<0.0001^c^
PR3-ANCA ELISA	57/244 (23.4%)	51/222 (23.0%)	6/22 (27.3%)	6/254 (2.7%)	5/65 (7.7%)	0/91 (0%)	<0.0001^a^
							0.005^b^
							<0.0001^c^
aANCA IIF (Titer 1∶20)	101/244	89/222 (40.1%)	12/22 (54.5%)	51/254 (20.1%)	26/65 (40%)	10/91 (11%)	<0.0001^a^
							0.955^b^
	(41.4%)						<0.0001^c^

a p value for PSC vs. all controls.

b p value for PSC vs. AIH.

c p value for PSC vs. PBC.

### Comparison of PR3-ANCA and Established Assays

The sensitivity and specificity for PSC were 38.5% and 89.4%, respectively when compared to all controls by CIA. When compared to AIH the specificity was 78.5% and to PBC was 86.8%. The likelihood ratios (LR+/LR-) were 3.62/0.69 for PSC vs. all controls, 1.79/0.78 for PSC vs. AIH, and 2.92/0.71 for PSC vs. PBC. By ELISA, the sensitivity and specificity in PSC were 23.4% and 97.6%, respectively when compared to all controls. When compared to AIH the specificity was 92.3% and to PBC was 100%. The LR+/LR- were 9.89/0.78 for PSC vs. all controls, 3.04/083 for PSC vs. AIH, and +∞/0.77 for PSC vs. PBC.

Measurement of aANCA by IIF had a sensitivity and specificity of 41.4% and 83.9% respectively when PSC was compared to all controls. When compared to AIH the specificity was 60.0% and to PBC was 89.0%. The LR+/LR- were 2.56/0.70 for PSC vs. all controls and 1.03/0.98 for PSC vs. AIH, and 3.77/0.66 for PSC vs. PBC (Table 2).

**Table 2:** Sensitivity, specificity and likelihood ratios (LR+/LR-) in PSC when compared to controls.

	Sensitivity (95% CI)	Specificity	Specificity	Specificity	LR+/LR-	LR+/LR-	LR+/LR-
		PSC vs. All controls	PSC vs. AIH	PSC vs. PBC	PSC vs. All Controls	PSC vs. AIH	PSC vs. PBC
		(95% CI)	(95% CI)	(95% CI)			
PR3 CIA	38.50%	89.40%	78.50%	86.80%	3.62/0.69	1.79/0.78	2.92/0.71
	(32.4–44.9)	(84.9–92.9)	(66.5–87.7)	(78.1–93.0)			
PR3 ELISA	23.40%	97.60%	92.30%	100%	9.89/0.78	3.04/0.83	+∞/0.77
	(18.2–29.2)	(94.9–99.1)	(83.0–97.5)	(96.0–100)			
aANCA IIF	41.40%	83.90%	60.00%	89.00%	2.56/0.70	1.03/0.98	3.77/0.66
	(35.1–47.9)	(78.7–88.2)	(47.1–72.0)	(80.7–94.6)			

**Figure 1:**
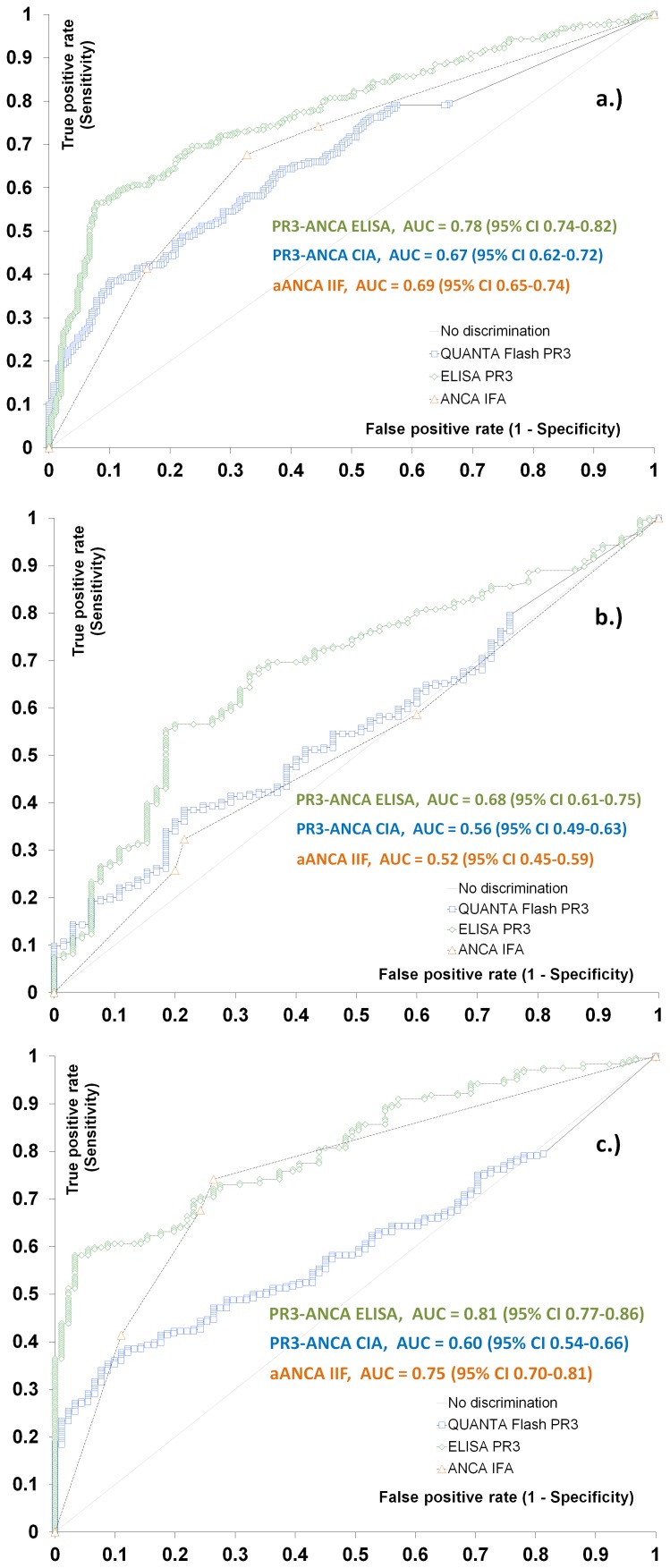
Comparative receiver operating characteristic (ROC) curves analysis. Comparative ROC analysis is shown for PR3-ANCA by ELISA, chemiluminescence immunoassay (CIA) and ANCA by indirect immunofluorescence (pANCA). Primary sclerosing cholangitis (PSC) vs. all liver controls (a) and vs. autoimmune hepatitis (AIH) (b), and vs. primary biliary cirrhosis (PBC) (c). Area under the curve (AUC) and 95% Confidence Intervals (CI) values for the individual assays are presented in the figure.

ROC curves were used to test the ability of the serological tests to discriminate PSC from other liver controls (Figure 1). When PSC was compared to all controls the area under the curve (AUC) values were 0.67 (CIA), 0.78 (ELISA), and 0.69 (IIF), respectively. When PSC was compared to AIH the area under the curve (AUC) values were 0.56 (CIA), 0.68 (ELISA), and 0.52 (IIF), respectively. When PSC was compared to PBC the area under the curve (AUC) values were 0.60 (CIA), 0.81 (ELISA), and 0.75 (IIF), respectively.

**Figure 2:**
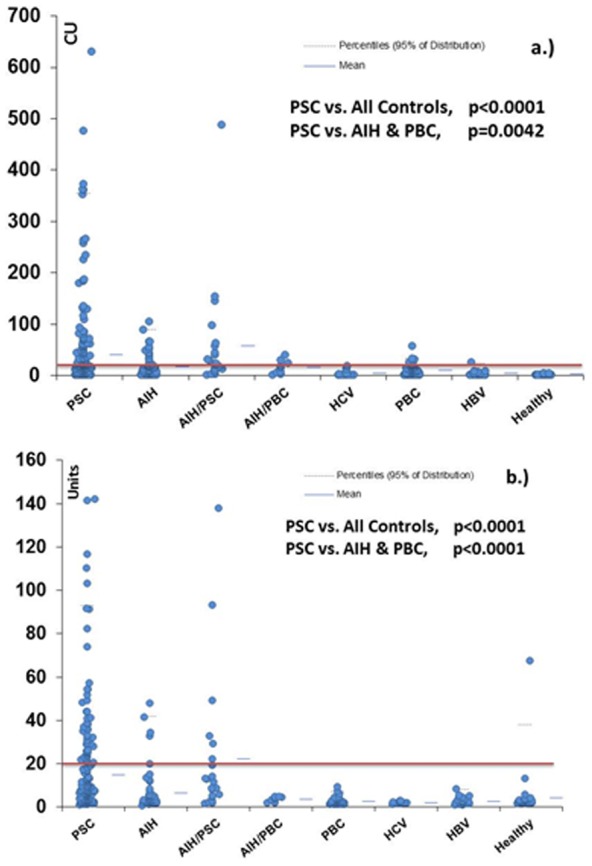
Comparative descriptive analysis. PR3-ANCA in primary sclerosing cholangitis (PSC) and various pathological and healthy controls (a. PR3-ANCA chemiluminescence immunoassay (CIA), b. PR3-ANCA ELISA). The recommended cut-off by the manufacturer is 20 chemiluminescent units (CU). Groups are as follows: autoimmune hepatitis (AIH); primary biliary cirrhosis (PBC); AIH-PSC overlap; AIH-PBC overlap; hepatitis C virus (HCV) infection; hepatitis B virus (HBV) infection; healthy controls.

Mean titers of PR3-ANCA CIA were significantly higher in PSC (PSC 39.9CU, AIH-PSC 58.7CU) compared to other liver cohorts (p<0.0001): AIH 17.3 CU, AIH-PBC 15.6 CU, PBC 9.8 CU, HCV 4.7 CU, HBV 4.2 CU, healthy controls 2.5 CU. Mean titers of PR3-ANCA ELISA were significantly higher in PSC (PSC 14.9 CU, AIH-PSC 22.3) compared to controls (p<0.0001): AIH 6.4 CU, AIH-PBC 3.7 CU, PBC 2.7 CU, HCV 2.1 CU, HBV 2.7 CU, healthy controls 4.4 CU (Figure 2).

### Clinical parameters of PSC patients

The available clinical and biochemical parameters of the PSC patients are reported in Table 3. PR3-ANCA positivity by CIA was associated with younger age (p<0000.1), higher ALT (p = 0.0002), AST (p<0.0001), ALP (p<0.0001), and platelet levels (p = 0.0076). PR3-ANCA did not significantly distinguish between sex, disease duration, small duct PSC, cholangiocarcinoma, cirrhosis, esophageal varices, ascites, hepatic encephalopathy, or UDCA use. Due to the lower frequency of PR3-ANCA measured by ELISA, only CIA results were analyzed for clinical parameters.

**Table 3:** Comparison of the clinical parameters of PSC patients at the time of serum testing according to the PR3-ANCA status as measured by chemiluminescence immunoassay (CIA).

	PSC patients	PR3-ANCA positive	PR3-ANCA negative	p value
Age, years (range) (n)	45.3 (18–86) (n = 218)	39.4 (19–78) (n = 78)	48.6 (18–86) (n = 140)	<0.0001^a^
Sex, male (%)/female (%)	133 (61%)/85 (39%)	55 (71%)/23 (29%)	78 (56%)/62 (44%)	0.067^b^
ALT, U/L (range) (n)	91 (8–576) (n = 201)	133 (8–576) (n = 74)	66 (12–294) (n = 127)	<0.0001 a
AST, U/L (range) (n)	74 (17–730) (n = 198)	109 (17–730) (n = 73)	55 (18–247) (n = 125)	<0.0001 a
ALP, U/L (range) (n)	273 (41–1959) (n = 202)	352 (52–1959) (n = 74)	228 (41–1349) (n = 128)	<0.0001 a
Total bilirubin, mg/dL (range) (n)	22 (3–445) (n = 202)	25 (5–340) (n = 74)	20 (3–445) (n = 128)	0.12 a
INR (range) (n)	1.1 (0.9–2.4) (n = 194)	1.1 (0.9–2.0) (n = 71)	1.1 (0.9–2.4) (n = 123)	0.08 a
Albumin, g/L (range) (n)	41 (21–54) (n = 78)	41 (28–50) (n = 31)	41 (21–54) (n = 47)	0.98 a
Hemoglobin g/L (range) (n)	135 (79–167) (n = 204)	133 (89–167) (n = 75)	137 (79–167) (n = 129)	0.18 a
Platelet count ×10^9^/L (range) (n)	266 (37–608) (n = 185)	288 (98–608) (n = 64)	253 (37–589) (n = 121)	0.0076 a
Creatinine µmol/L (range) (n)	78 (50–127) (n = 29)	76 (60–102) (n = 9)	79 (50–127) (n = 20)	0.91 a
Disease duration, years (range) (n)	7.8 (0–27) (n = 201)	7.4 (0–25) (n = 72)	8.0 (0–27) (n = 129)	0.73 a
Small duct PSC, n (%)	28/202 (14%)	11/28 (39%)	17/28 (61%)	0.9 b
Cholangiocarcinoma, n (%)	5/167 (3%)	3/5 (60%)	2/5 (40%)	0.3 b
Cirrhosis, n (%)	34/74 (46%)	16/34 (47%)	18/34 (53%)	0.21 b
Ursodeoxycholic Acid (UDCA) use, n (%)	64/87 (74%)	27/64 (42%)	37/64 (58%)	0.82 b
Varices, n (%)	15/52 (29%)	7/15 (47%)	8/15 (53%)	0.51 b
Ascites, n (%)	12/55 (22%)	7/12 (58%)	5/12 (42%)	0.15 b
Hepatic encephalopathy, n (%)	5/54 (9%)	3/5 (60%)	2/5 (40%)	0.58 b

ALP, alkaline phosphatase; ALT, alanine aminotransferase; AST, aspartate aminotransferase, INR, international normalized ratio; UDCA, ursodeoxycholic acid.

a Mann-Whitney test was used for continuous variables.

b Student's t-test was used for qualitative measurements.

### Clinical associations of PR3-ANCA in PSC and IBD

The clinical association of IBD in the PSC patients was available in 204/244 (83.6%) of the cohort. 62/244 (25.4%) had no associated IBD, 27/242 (11.1%) had Crohn's disease (PSC-CD), 111/244 (45.5%) had UC (PSC-UC) and 4/244 (1.6%) had IBD-unclassified (PSC-IBD-U). By CIA, 58/142 (40.8%) of the PSC & IBD patients [PSC-CD 13/27 (48.1%), PSC-UC 45/111 (40.5%), PSC-IBD-U 0/4] were positive for PR3-ANCA compared to 19/62 (30.6%) of the PSC with no associated IBD patients (p = 0.22). By ELISA, 38/142 (26.8%) of the PSC & IBD patients [PSC-CD 6/27 (22.2%), PSC-UC 32/111 (28.8%), PSC-IBD-U 0/4] were positive for PR3-ANCA compared to 10/62 (16.1%) of the PSC & no associated IBD patients (p = 0.14). aANCA by IIF were detected in 58/142 (40.8%) of the PSC & IBD patients [PSC-CD 7/27 (25.9%), PSC-UC 47/111 (25.9%), PSC-IBD-U 4/4 (100%)] compared to 26/62 (41.9%) of the PSC & no associated IBD patients (p = 1) (Table 4). There was no significant difference between any of the tests when PSC-CD was compared to PSC-UC.

**Table 4:** Frequency of PR3-ANCA in PSC & IBD subgroups.

	PSC & IBD	PSC-CD	PSC-UC	PSC-IBD-U	PSC & no IBD	p value
PR3-ANCA CIA	58/142 (40.8%)	13/27 (48.1%)	45/111 (40.5%)	0/4	19/62	0.22^a^
				0%	-30.60%	1.00 b
PR3-ANCA ELISA	38/142 (26.7%)	6/27 (22.2%)	32/111 (28.8%)	0/4	Oct-62	0.14 a
				0%	−16.10%	0.41 b
aANCA IIF	58/142 (40.8%)	7/27 (25.9%)	47/111 (42.3%)	4-Apr	26/62	1.00 a
				−100%	−41.90%	0.64^b^

a P value calculated between PSC & IBD and PSC & no IBD.

b P value calculated between PSC-CD and PSC-UC.

## Discussion

PR3-ANCA is an extensively described diagnostic and prognostic serological biomarker for primary systemic vasculitis [23]. More recently, PR3-ANCA, when measured by a novel CIA platform, has been described in a significant proportion of IBD patients, specifically UC, where it was related to disease severity [18], [24]. Because PSC is commonly associated with IBD [25], we were interested to determine if PR3-ANCA is also a biomarker of this condition. In this study we found that the frequency of PR3-ANCA when measured by CIA is 38.5% in PSC patient sera and only 10.6% in liver disease controls (p<0.0001). When measured by ELISA, the prevalence of PR3-ANCA in PSC is lower at 23.3%; however, it had a higher specificity compared to CIA (97.6% vs. 89.4%). PR3-ANCA measured by both CIA and ELISA were more specific than the IIF aANCA (83.9%), which is a traditional serological marker of PSC [26]. These results are clinically and diagnostically important as the IIF testing for aANCA is associated with a number of limitations. For example, IIF assays are time consuming, observer-dependent, low throughput, and require highly trained personnel. In addition, the standardization of ELISA has proven challenging largely because of inter-manufacturer variation of ANCA detection kits [27]–[29]. Taken together, despite international consensus and guidelines for ANCA testing [7], [30], these factors still generate significant inter-laboratory variation of results [31], [32]. Although aANCA is currently used as a biomarker for PSC, it also has significant overlap with other disorders including AIH and UC. Our study has shown that PR3-ANCA performs better than aANCA for the diagnosis of PSC and avoids the challenges associated with IIF testing.

Patients presenting with elevated liver enzymes require a full diagnostic workup and the differential diagnosis of potential hepatopathies is wide. The present data suggest that the measurement of PR3-ANCA are a useful biomarker to aid in the differentiation of PSC and other liver diseases including AIH, PBC. Other serological biomarkers are used for the diagnosis of AIH (ANA, F-actin, ASMA [33]) and PBC (i.e. AMA, gp210, sp100) [34], [35], however, apart from aANCA by IIF, which can also be seen in AIH, no simple serological test is currently available to alert the clinician to possible PSC. With multiplex assays becoming more common [36], [37], perhaps the addition of PR3-ANCA to a liver panel should be considered: given that PSC is rare and not encountered frequently by many clinicians, such a blood result could initiate further investigation, such as a MRCP, in a more prompt way outside of specialist centers.

In our study, PR3-ANCA in PSC was associated with higher levels of hepatocellular (ALT, AST) and cholestatic (ALP) liver enzymes. Although there was no statistically significant difference between PR3-ANCA in disease severity or progression as measured by the presence of cirrhosis, esophageal varices, hepatic encephalopathy, or ascites, the relative number of these patients in the cohort was low. Additionally, the ability of PR3-ANCA to predict small duct PSC or the development of cholangiocarcinoma was limited by small sample numbers. However, the finding that PR3-ANCA is associated with patients with higher liver enzymes may represent a subgroup of patients with a more inflammatory phenotype who potentially may respond to future anti-inflammatory compounds. Larger prospective studies will be needed to further identify the clinical association of PR3-ANCA in PSC, including identifying subgroups of patients and response to medications.

Our data also demonstrate that PR3-ANCA in PSC is not exclusively related to underlying IBD. Due to differences in data collection between the clinical sites, the IBD status was only known in 83.6%. Despite this, there was no significant difference between PR3-ANCA in PSC-CD, PSC-UC, PSC-IBD-U, or PSC without associated IBD. However, PR3-ANCA may be a marker of PSC in CD. Previous studies have shown that the frequency of PR3-ANCA in CD is low (i.e. <2%) [18], [38], however these studies did not include patients with PSC. Our study found that 48.1% of PSC-CD patients had PR3-ANCA when measured by CIA. Although larger studies are needed to confirm this association, if PR3-ANCA is seen in CD, it may predict and/or prompt the clinician to evaluate the patient for associated PSC.

We acknowledge limitations of our study including the fact that it is retrospective and lacks the serial measurements of PR3-ANCA during the disease course. Although the diagnosis of PSC was made by experts in the field, due to the multi-centre nature of the study, a lack of a standardized diagnostic approach, is a limitation. Although the specificity of PR3-ANCA in PSC is high, we acknowledge that the sensitivity of the test is very low. At this point we are not recommending the use of this test to solely rule out the diagnosis of PSC. Additional studies are warranted to investigate if PR3-ANCA in PSC is associated with disease prognosis or response to medications and if PR3-ANCA in CD may indicate an increased likelihood of the presence of PSC in these patients.

## Conclusions

Our data demonstrate that PR3-ANCA can be detected in a significant proportion of PSC patients and are a specific biomarker in the context of other liver disorders including autoimmune liver diseases. PR3-ANCA in PSC was found to be associated with higher liver enzymes and may represent a more inflammatory subtype of disease. PR3-ANCA in PSC do not seem to be related to a co-diagnosis of IBD and more studies are needed to determine if it may be a biomarker of PSC in CD.
